# Nerve Regeneration after a Nerve Graft in a Rat Model: The Effectiveness of Fibrin Glue

**DOI:** 10.3390/jpm14050445

**Published:** 2024-04-24

**Authors:** Giovanni Zabbia, Francesca Toia, Federico Coppola, Giovanni Cassata, Luca Cicero, Giuseppe Giglia, Roberto Puleio, Adriana Cordova

**Affiliations:** 1 Plastic and Reconstructive Surgery, Department of Precision Medicine in Medical, Surgical and Critical Care, University of Palermo, Via del Vespro 129, 90127 Palermo, Italy; giovanni.zabbia@policlinico.pa.it (G.Z.); francesca.toia@unipa.it (F.T.); adriana.cordova@unipa.it (A.C.); 2 Centro Mediterraneo Ricerca e Training (Ce.Me.Ri.T), Istituto Zooprofilattico Sperimentale della Sicilia “A. Mirri”, 90129 Palermo, Italy; giovanni.cassata@izssicilia.it (G.C.); luca.cicero@izssicilia.it (L.C.); 3 Department of Biomedicine, Neuroscience and Advanced Diagnostics (BiND), Section of Human Physiology, University of Palermo, 90127 Palermo, Italy; giuseppe.giglia@unipa.it; 4 Laboratorio Istopatologia e Immunoistochimica, Dipartimento Ricerca Biotecnologica e Diagnostica Specialistica, Istituto Zooprofilattico Sperimentale della Sicilia “A. Mirri”, 90129 Palermo, Italy; roberto.puleo@izssicilia.it

**Keywords:** nerve injury, nerve regeneration, nerve repair, fibrin glue, peripheral nerve injuries

## Abstract

Background: Simulating the post-traumatic continuity defect of small human peripheral nerves, we compared the effectiveness of fibrin glue with neurorrhaphy for nerve gap restoration. Methods: In twenty-four male Wistar rats, a fifteen mm defect in one sciatic nerve only was made and immediately repaired with an inverted polarity autograft. According to the used technique, rats were divided into Group A (Control), using traditional neurorrhaphy, and Group B (Study), using fibrine glue sealing; in total, 50% of rats were sacrificed at 16 weeks and 50% at 21 weeks. Before sacrifice, an assessment of motor function was done through Walking Track Analysis and an electroneurophysiological evaluation. After sacrifice, selected muscle mass indexes and the histology of the regenerated nerves were assessed. All data were evaluated by Student’s *t* test for unpaired data. Results: No significant differences were found between the two groups, with only the exception of a relative improvement in the tibialis anterior muscle’s number of motor units in the study group. Conclusion: Despite the fact that the use of fibrin glue as a nerve sealant is not superior in terms of functional recovery, its effectiveness is comparable to that of microsurgical repair. Hence, the faster and technically easier glueing technique could deserve broader clinical application.

## 1. Introduction

Peripheral nerve injuries still represent a serious cause of disability; despite the progress made in microsurgical techniques and materials [1,2], it is often difficult to obtain a complete recovery of the axons and, therefore, of the function of the innervated muscles.

In case of injury with a nerve defect, the current gold standard is the grafting of an autologous sensory nerve segment between the two nerve stumps [3,4,5]. The standard method used to reconnect the stumps is a microsurgical tensionless suture [6,7,8].

This technique, despite being the current standard treatment, has several limitations; in fact, a microsurgical suture can be a time-consuming and technically intricate process, particularly in challenging situations with small-diameter nerves, requiring appropriate surgical skills [9,10,11]. Moreover, the use of sutures in nerve repair might not guarantee complete neural regeneration due to tissue responses such as inflammation and scarring at the repair site [12,13], phenomena of Wallerian degeneration [14] and the possible occurrence of a neuroma at the site of anastomosis due to a granulomatous reaction [15,16]. Finally, the potential disruption of axonal regeneration quality may occur due to the compromise of the intraneural vascular supply resulting from the tension induced by the knot-tying of sutures [17,18].

Fibrin glue is a solution of fibrinogen, thrombin and sealing proteins that could find applications in nerve repair. When these proteins mix during application, they form a clot at the intervention point, simulating the final stage of the coagulation cascade. They are currently used to fix skin grafts (for example, on burn wounds) and to facilitate flap adhesion to the receiving site after extensive surgical dissections. Their composition allows for immediate use without the need for preventive treatments [19,20].

Fibrin glue is widely utilized in peripheral nerve surgery due to its simplicity, reduced tissue manipulation, diminished suture necessity, and shorter procedural durations. However, its application is still considered off label, as the most essential question remains whether fibrin glue also results in similar nerve recovery [21,22,23].

The aim of this study was to evaluate the effectiveness of the use of fibrin glue (Tisseel^®^) in securing a nerve graft without the use of sutures and compare the outcomes to those of traditional microsurgical repair. This method could reduce the suture line inflammation, and therefore scarring, improving axonal regeneration and accelerating the time of functional recovery [24,25,26].

## 2. Materials and Methods

The experimental protocol on the animals was conducted in accordance with the D.L. number 26 of 14 March 2014, authorization number 576/2015-PR, 23 June 2015, on the protection of animals used for experimental purposes or for other scientific purposes. Surgical procedures and animal housing took place at the Experimental Zooprophylactic Institute of Sicily. 

Power analysis determined that 10 animals per group provided 80% statistical power with an alpha of 0.05, based on an expected 20% effect on the Sciatic Functional Index (SFI). Twenty-four adult male Wistar rats weighing between 250 g and 450 g were used to account for eventual animal loss. Rats were housed in a temperature- and humidity-controlled room with 12–12 h light/dark cycles, and fed standard chow and water ad libitum. Measures were taken to minimize pain and discomfort, taking into account human endpoints for animal suffering and distress.

All surgical procedures were performed by the same operator and only on one limb of the animal, to allow for a greater degree of mobility and self-sufficiency. In preparation for surgery, rats were anesthetized first with Isofluorane for induction, followed by the intraperitoneal administration of 1 mL/kg of a Ketamine/xylazine cocktail (91 mg/mL Ketamine + 9.1 mg/mL Xylazine).

**Figure 1:**
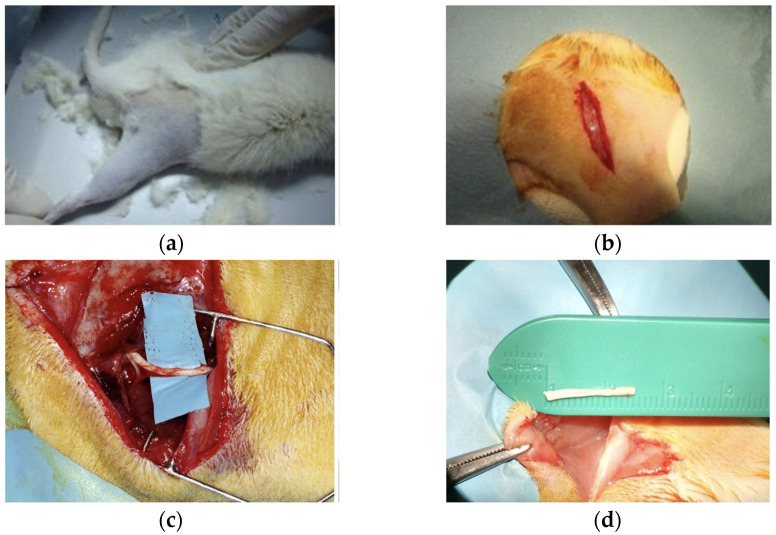
(**a**) Trichotomy of the right rear limb; (**b**) skin incision of 40 mm; (**c**) dissection and exposure of the sciatic nerve; (**d**) nerve section and autograft obtained.

Following anesthesia, right rear limb trichotomy and disinfection were performed (right rear limb) (Figure 1a); the animal was positioned in a prone position with the limbs fixed in abduction. The operation was performed under aseptic conditions and using a PowerFocus surgical microscope. The sciatic nerve was isolated through a skin incision of 40 mm, beginning 0.5 cm lateral to the dorsal midline of the rat and extending by 4 cm along the tibio-femoral articulation; subsequent detachment and retraction of the gluteus and bicep femoris muscles exposed the underlying sciatic nerve (Figure 1b,c). 

Each nerve was dissected first proximally (approximately 10 mm from its emergence from the sciatic notch) and then distally, before division into its terminal branches and the creation of a defect of 15 mm. (Figure 1d). The animals were randomly assigned to two groups, A (*n* = 12, Control Group) and B (*n* = 12, Study Group), according to the repair method. In both groups, the repair was performed by an inverted polarity autograft, using the same segment derived from the nerve section as the graft donor.

**Figure 2:**
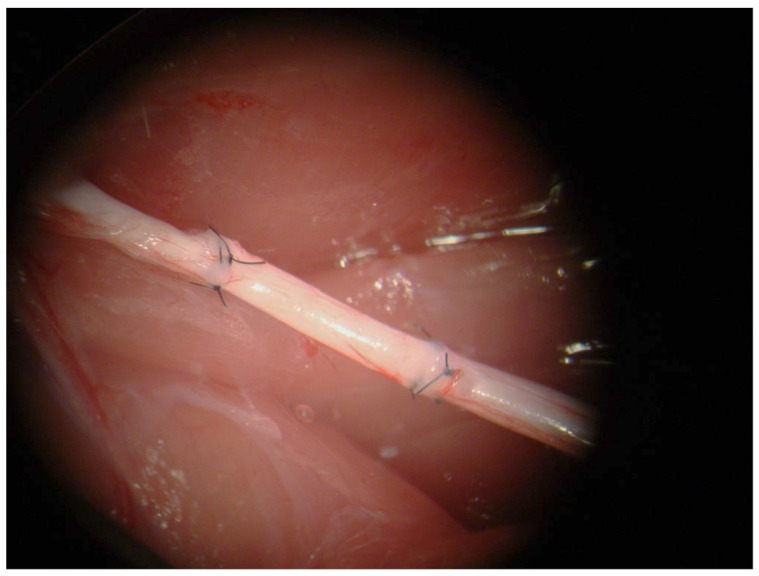
Nerve autograft with inverted polarity fixed with three epi-perineural sutures.

In Group A (Control group), the segment was fixed, both proximally and distally, by 3 single-stitch equidistant epi-perineural sutures, (Ethilon 9/0) under microscope magnification (Figure 2).

**Figure 3:**
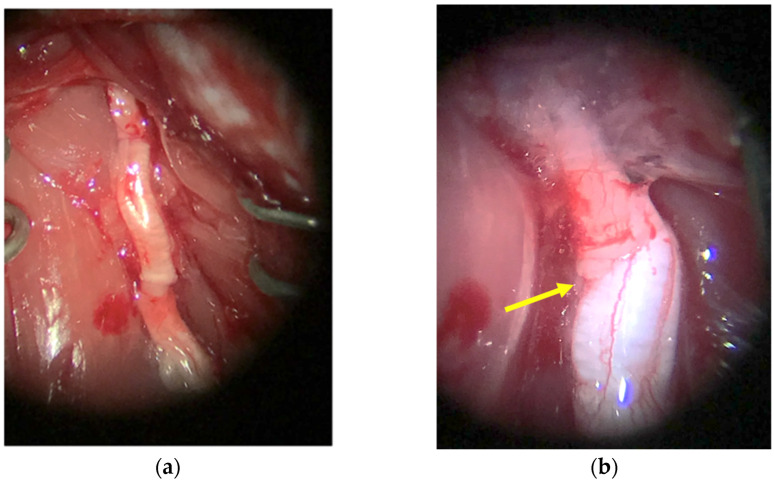
(**a**) Nerve autograft with inverted polarity fixed with fibrin glue; (**b**) particular of the suture line with fibrin glue (the yellow arrow pinpoints the specific location of the suture line where fibrin glue was applied).

In Group B (Study group), the segment was fixed, both proximally and distally, by Tisseel^®^ in the epineurium of the proximal and the distal stumps (Figure 3).

In both groups, the muscular and fascial layers were subsequently closed by resorbable sutures with 4/0 threads, and the skin by a continuous suture with 4/0 thread, after careful hemostasis.

At the end of the surgical procedures, 2 mL of NaCl fluid therapy and Enrofloxacin (10 mg/kg sc) were administered intraperitoneally to prevent the dehydration of the animal and for prophylactic antibiotic purpose.

To awake the animal, Atipamezole Hydrochloride 1 mL/kg was given intramuscularly.

Each animal was then transferred to a cage and assigned an identification number.

Rats were sacrificed at the 16th week or 21st week. 

For both the control and the study group, 50% of rats were sacrificed at 16 weeks and 50% at 21 weeks. Before sacrifice, all rats underwent Walking Track Analysis (WTA) for the evaluation of motor function, and electroneurophysiological evaluation. After sacrifice, the calculation of muscle mass indexes was performed, specifically the Gastrocnemius Muscle Weight Ratio (GMWR) and Tibialis Anterior Muscle Weight Ratio (TAMWR); additionally, a histological examination of the regenerated nerve was performed.

All data collected in the Study Group and Control Group populations were evaluated by Student’s *t* test for unpaired data, considering significant each value with *p* < 0.05.

### 2.1. Walking Track Analysis

For the functional evaluation, the WTA was conducted with the Sciatic Functional Index (*SFI*) measurement. The rats were tested on a straight path of 75 cm, after coloring the hind legs with black ink to impress the footprints on a strip of white paper placed on the floor of the path. From the print obtained, the following measurements were extrapolated: Print Length (*PL*), the distance between the heel and the third finger; Toe Spread (*TS*), the distance between the first and the fifth finger; and Intermediary Toe Spread (*ITS*), the distance between the second and the fourth finger. The formula used, a reinterpretation made by Bain in 1989, is as follows:

$$SFI=-38.3\;\left(\frac{EPL-NPL}{NPL}\right)+109.5\left(\frac{ETS-NTS}{NTS}\right)+13.3\left(\frac{EIT-NIT}{NIT}\right)-8.8$$

The letters *E* and *N* stand, respectively, for Experimental (operative limb) and Normal (healthy limb). The index values range from 0 to −100, 0 indicating a normal situation, and 100 a complete sciatic nerve injury.

### 2.2. Electroneurophysiological Evaluation

Measurements were detected in the Gastrocnemius and Tibialis Anterior muscles of each operated limb, using the contralateral as a control. Monopolar needle electrodes were placed in each muscle at a fixed distance from each other. The registration of the Compound Muscle Action Potential (CMAP) was made using a pair of monopolar needle electrodes, applied according to the “belly tendon” assembly. The reference electrode was inserted into the subcutis of a front limb. The stimulation was performed by a single pulse with a duration of 0.1 ms, with a diode-square wave; the amplitude was measured peak-to-peak. Using the Motor Unit Number Estimation (MUNE) technique, an estimate of the number of motor units that make up a muscle was then made, dividing the amplitude of the CMAP by the mean amplitude of the individual motor unit potentials (Single-Motor Unit Action Potential: SMUAP).

### 2.3. Muscle Mass Indexes

**Figure 4:**
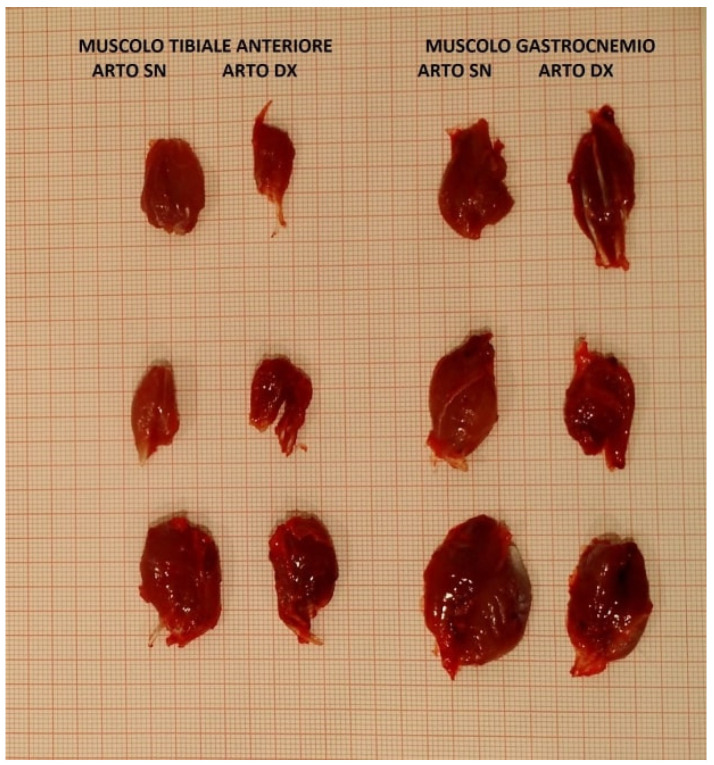
Tibialis Anterior Muscle and Gastrocnemius Muscle; example obtained muscle.

After the sacrifice, the Gastrocnemius and Tibialis Anterior muscles of both hind limbs were taken in full (Figure 4) and weighed using an electronic precision balance.

The Muscle Weight Ratio (MWR) was then calculated for each muscle, relying on the comparison between the values obtained from the measurement of the muscles of the operated limb (right) and the healthy limb (left); to do this, the following formulas were used:

$$GMWR=\frac{Weight\;of\;the\;reinnervated\;gastrocnemius}{Weight\;of\;the\;controlateral\;gastrocnemius}$$

$$TAMWR=\frac{Weight\;of\;the\;reinnervated\;tibialis\;anterior}{Weight\;of\;the\;controlateral\;tibialis\;anterior}$$

### 2.4. Histological Analysis

The sciatic nerves of both lower limbs were taken proximally to the proximal suture and distally to the distal suture, fixed in 4% paraformaldehyde in a saline phosphate buffer (PBS) for 2–4 h and then washed and stored in 0.2 g of glycine in 100 mL of PBS before inclusion. After washing in PBS for a few minutes, the nerves were immersed for 4 h in a 2% solution of osmium tetroxide and then dehydrated in a growing series of alcohol.

The samples were then included in paraffin and cross sections were obtained with a thickness of 3–5 μm. By means of an optical microscope connected to a video camera, images at 100× magnification were acquired.

An area of 5500 µm^2^ was then selected (as representative of the entire section of the nerve), distally to the distal suture, for the calculation of the parameters useful for the evaluation of the degree of nerve regeneration of the samples.

The parameters evaluated for morphometric analysis of the nerve were as follows:

- n = Number of myelinated fibers;
- Fiber Area (FA): Area occupied by nerve fibers (μm^2^) on an assessed sample area of 5500 μm^2^;
- Fiber Density (FD): Number of myelinated fibers (n) over the entire area of the sciatic nerve section (μm^2^).

## 3. Results

Four rats (two from the Study Group and two from the Control Group) were excluded from the study as they died soon after surgery. Three other rats (2 of the Study Group and 1 of the Control Group) presented self-mutilation phenomena after the 16th week and were excluded from the functional evaluation at 21 weeks.

### 3.1. Walking Track Analysis

Walking Track Analysis did not show any statistically significant differences between the two groups at both 16 and 21 weeks (*p* = 0.8 and *p* = 0.9, respectively) (Table 1).

### 3.2. Electroneurophysiological Evaluation

**Figure 5:**
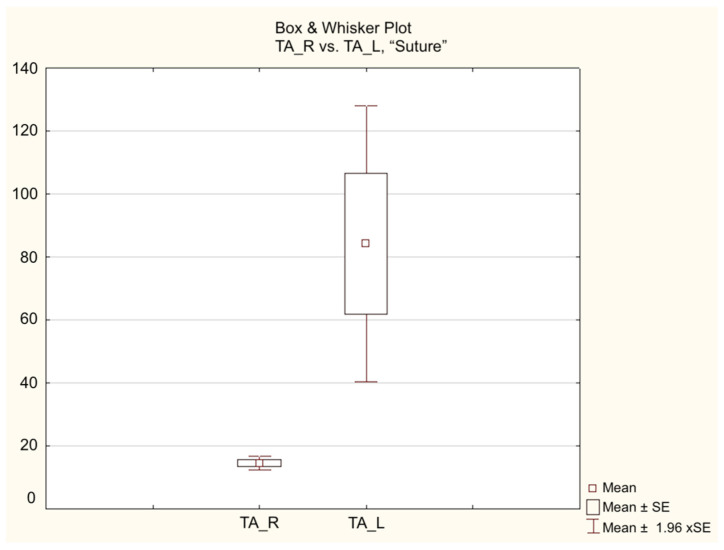
Results of electroneurophysiological evaluation in the 21-week Control Group.

**Figure 6:**
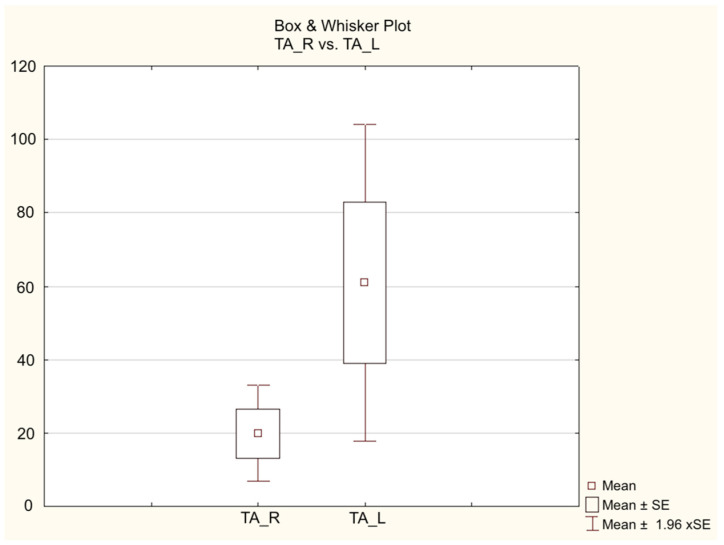
Results of electroneurophysiological evaluation in the 21-week Study Group.

Intragroup analysis did not show any significant differences for the Gastrocnemius muscle in either group, both at 16 and at 21 weeks. However, at both 16 and at 21 weeks, there was a significant difference in the Tibialis Anterior muscle only in the Control Group (*p* = 0.01), with no significant difference in the Study Group (*p* = 0.1) (Figure 5 and Figure 6).

### 3.3. Muscle Mass Indexes

#### 3.3.1. GMWR

A detailed summary of the GMWR and Gastrocnemius average weight (of each limb) in both the Control and Study Groups at 16 and 21 weeks after surgery is shown in Table 2.

Statistical analysis did not show any significant differences in GMWR between the Control and Study Groups at either 16 or 21 weeks.

#### 3.3.2. TAMWR

A detailed summary of the TAMWR and tibialis anterior average weight (of each limb) of both the Control and Study Groups at 16 and 21 weeks is shown in Table 2.

Student’s *t* test between the Control Group and Study Group in the population did not show any significant differences in the TAWR at either 16 or 21 weeks post-surgery (t = 1.0037; df = 8; *p* = 0.3449 and t = 1.1142; df = 8; *p* = 0.2975, respectively).

### 3.4. Histological Analysis

**Figure 7:**
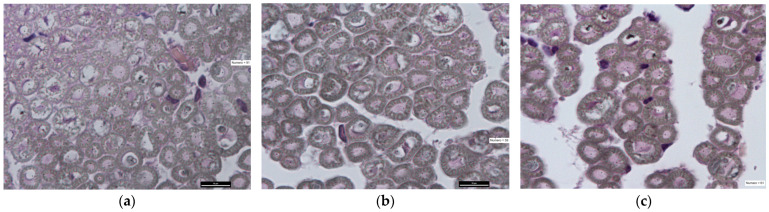
Section of colored nerve with Osmium tetroxide, 100× magnification. (**a**) Healthy nerve, n = 91; (**b**) nerve Control Group at 16 weeks, n = 59; (**c**) nerve Study Group at 16 weeks, n = 61.

From the histological evaluation (Figure 7), the following parameters were detected: average number of regenerated nervous fibers, fiber area evaluation and fiber density at 16 and 21 weeks after surgery in both the Control and Study Groups.

Detailed aforementioned values of both the Control and Study Groups at 16 and 21 weeks are shown in Table 3.

Statistical analysis did not show any significant difference in regenerated nerve fibers, fiber area or fiber density between the Control and Study Group at either 16 or 21 weeks.

Student’s *t* test between the Control Group and Study Group in the population did not show any significant differences at either 16 and 21 weeks post-surgery in the regenerated nervous fibers (t = 0.73; df = 8; *p* = 0.486 and t = 0.94; df = 8; *p* = 0.3734, respectively), in the fiber area (t = 1.85; df = 8; *p* = 0.1 and t = 2.144; df = 8; *p* = 0.0647, respectively), nor in the fiber density (t = 1.0476; df = 8; *p* = 0.3254 and t = 1.04; df = 8; *p* = 0.33, respectively). 

## 4. Discussion

The use of fibrin glue in the repair of peripheral nerves is not a new practice, with the first reports of its use in the literature dating back to the 1940s. Despite these early reports, the use of fibrin glue as a sealant for peripheral nerve injury was quite uncommon, due to low tensile strength and rapid absorption.

During the 1970s, Matras and colleagues created a more concentrated formula that enhanced its longevity [27,28] with the marketing of the first Tisseel^®^ preparation.

Despite Tisseel^®^ being introduced more than four decades ago, there is still limited literature on its application and effectiveness in repairing peripheral nerves, with most studies focusing on its efficacy in rat models [9,10,12,21,29,30,31,32,33,34,35,36,37,38,39,40]. This could be attributed to two main factors. Firstly, its approval by the U.S. Food and Drug Administration in 1998 was relatively recent, and secondly, the use of Tisseel^®^ for nerve repair was firstly considered off label. In fact, at the beginning, Tisseel^®^ was only officially approved as an adjunct in maintaining hemostasis during cardio-pulmonary bypass and as a sealant during temporary colostomies [41].

For these two reasons, although lots of animal studies have been carried out to evaluate the effectiveness of fibrin glue in the coaptation and healing of peripheral nerve injuries, the literature still lacks controlled human trials comparing fibrin sealants and suturing techniques [42,43].

Several experimental animal studies conducted in the past years have shown variable outcomes; in some cases, the result of the use of fibrin glue was superimposable to that of the sutures [29,30,31], if not even better [9,10,32,33,34,35], while in other cases it seemed to be worse [12,44,45].

A few systematic reviews were carried out more recently, with the final result of no substantial difference between the two repair methods in terms of nerve regeneration and motor function restorations.

However, most of the assessed studies were carried out on animal models, with the use of different types of glue and different types of evaluated parameters [9,10,12,21,29,30,31,32,33,34,35,36,37,38,39,40].

Fibrin glue works by simulating the end stage of the coagulation cascade, forming a substance resembling a physiological blood clot. This clot acts as a protective layer that holds the nerve stumps and protect them from the surrounding scar tissue, allowing nerve fibers to heal within the epineurium [27].

Menovsky and Beek’s study demonstrated more adhesions, fibrosis, and thickening at the repair site in their suture group [46], while InalöZ et al. demonstrated how nerve coaptation was superior in their fibrin glue group according to the electromyography results, neuroconduction studies, and histopathological examination [47].

In this study, the study group was compared to a population of overlapping characteristics (15 mm sciatic nerve gap) treated with the current gold standard of care for these lesions: a nerve autograft with reverse polarity fixed with sutures.

We evaluated the effectiveness of the fibrin glue repair technique, trying to overcome the well-known limits of injured nerve’s repair by suture, such as degenerative nerve bundles, excessive local inflammation, scar and the possibility of neuroma formation (or foreign body granuloma) at the anastomosis site.

Both groups were evaluated at 16 and 21 weeks.

For the evaluation of nerve regeneration, the following parameters were evaluated:

- Walking Track Analysis for the analysis of the locomotor activity;
- Electroneurography;
- Weight of the muscles (and their ratio);
- Histological examination of the regenerated.

The Walking Track Analysis is a useful tool to evaluate the functional restoration in the regeneration of peripheral nerves for the mouse and rat model. The test is based on the assumption that a better regeneration of the reconstructed sciatic nerve corresponds to better running performance [48]. The parameter used in the Walking Track Analysis was the SFI, for the calculation of which we used measurements obtained from the imprint left by the rat on a sheet of white paper placed on a straight path inside a tunnel.

The other evaluated parameters were more objective and bias-from-animal-behavior free.

The analysis of the muscle mass indexes evaluates the tropism of a muscle reinnervated by a reconstructed nerve. A denervated muscle undergoes atrophy, with a speed directly proportional to the muscle mass and denervation time. The reinnervation of the surviving fibers (within certain time limits) causes the degenerative phenomenon to stop and, progressively, the muscle to regain its trophism [49].

On this basis, the weight of the Tibialis Anterior and Gastrocnemius muscles, innervated by the branches of the sciatic nerve, represents a good indirect index for the evaluation of nerve regeneration [29,50,51].

Statistical analysis did not show any significant difference in all the aforementioned evaluated parameters (WTA, electroneurofisiological evaluations, muscle mass index and histological analysis) between the Control and the Study Group at either 16 or 21 weeks. The only significant difference (*p* = 0.01) was found in the electroneurophysiological analysis of the Tibialis Anterior muscles in the Control Group.

At the level of the Tibialis Anterior (at both 16 and 21 weeks), a significant decrease in the number of motor units estimated in the reinnervated muscle was found in the Control Group, compared to the contralateral (*p* = 0.01); in the Study Group, this reduction was not significant (*p* = ns) and there is, therefore, a likely improvement in the number of motor units compared to the group treated with the traditional suture.

This study was aimed at evaluating the effectiveness of the use of fibrin glue compared to the gold standard microsurgical repair technique with a suture. No statistically significant differences between the two analyzed groups emerged. We found an overlap in terms of the results of both the examined techniques which could be interchangeable in terms of post-injury nerve regeneration.

The only statistically significant result of this study is that the electroneurophysiological analysis of the Tibialis Anterior muscles in the Control Group (operated limb compared to healthy limb) pointed at a likely improvement in the number of motor units in the fibrin glue group compared to the current gold standard of care (microsurgical suture).

The fibrin glue technique of nerve repair has, from our perspective, many advantages compared to the microsurgical gold standard of care, the most important of which are the following: reduced surgical time, decreased fibrosis and inflammation, reduced induced trauma and neural scar tissue, better hemostasis and an easier stabilization of small grafts and, above all, technical ease of use.

Microsurgical repair, on the other hand, is a time-consuming and technically demanding procedure in cases with difficult exposure or small caliber nerves, and with many already outlined limitations. Besides, it requires a microsurgery-trained surgeon with experience in this field.

The fact that no statistically significant differences were found in the WTA, in the evaluation of muscle mass indexes and in the histological analysis can be linked to the sample size, or to the fact that the two techniques are interchangeable with overlapping results.

The results obtained encourage new research perspectives aimed at testing the use of fibrin glue on a larger sample to subsequently promote its use in clinical practice, considering that the results are superimposable to the standard technique, and there is an advantage of reduced suture time, and therefore costs.

Given fibrin glue’s advantages, particularly its technical ease of use, microsurgical suture limits, and the promising electroneurophysiological results, we think that, despite the unproved superiority of the glueing technique, the similar results can justify a broader use of the fibrin glue technique in clinical practice. This is based on the advantages listed above, primarily its being a less technically and time-demanding procedure compared to microsurgical repair.

Limitations of this study include the following:

- Sciatic Functional Index is a reference parameter used for evaluating the functional restoration in the regeneration of peripheral nerves for the mouse and rat model, but the results of this test can still be influenced by the behavior of the animal;
- Variability in fibrin glue preparation and usage; many fibrin glues exist, so this study’s results could be different using different glues commercially available;
- The sample size was relatively small, but due to ethical reasons was kept adherent to the preventive sample size calculation based on statistical power;
- We did not analyze, through histological staining, the potential to reduce the inflammation response when using fibrin glue compared to neurorrhaphy; we acknowledge that conducting such a study would be beneficial for our future investigations.

## 5. Conclusions

Our study demonstrates that the use of fibrin glue is an effective way to fix nerve autografts to the stumps of the injured peripheral nerve, as effective as microsurgical sutures.

From the evaluation of the number of motor units estimated in the Tibialis Anterior muscle, a statistically significant difference (*p* = 0.01) emerged, indicating a likely improvement in the number of motor units in the fibrin glue group, compared to the group treated with a traditional suture; the limited sample size, however, limits the significance of this superior result.

## Figures and Tables

**Table 1:** Average SFI (Sciatic Functional Index).

	16 Weeks Control Group	21 Weeks Control Group	16 Weeks Study Group	21 Weeks Study Group
Average SFI	−47.6 range −35/−60	−38 range −48/−19	−45.8 range −40/−68	−40 range −52/−20

**Table 2:** Average weight of the Gastrocnemius and Tibialis Anterior muscles. Average GMWR (Gastrocnemius muscle weight ratio) and Average TAMWR (Tibialis Anterior weight ratio).

		16 Weeks Control Group		21 Weeks Control Group		16 Weeks Study Group		21 Weeks Study Group	
Gastrocnemius muscles	Average weight	Operated Limb	Healthy Limb	Operated Limb	Healthy Limb	Operated Limb	Healthy Limb	Operated Limb	Healthy Limb
		1.008 g range: 0.41–1.38 g	1.712 g range: 1.06–2.2 g	0.7620 g range: 0.59–0.86 g	1.266 g range: 1.01–1.43 g	0.93 g range: 0.33–1.32 g	1.768 g range: 1.01–2.5 g	0.6460 g range: 0.31–0.84 g	1.2320 g range: 0.92–1.59 g
	Average GMWR	0.6041 range: 0.262820513–0.90566037		0.6368 range: 0.495798319–0.851485149		0.5027 range: 0.326732673–0.601990049		0.5253 range: 0.336956522–0.762376238	
Tibialis Anterior muscles	Average weight	OPERATED LIMB	HEALTHY LIMB	OPERATED LIMB	HEALTHY LIMB	OPERATED LIMB	HEALTHY LIMB	OPERATED LIMB	HEALTHY LIMB
		0.63 g range: 0.35–1.08 g	1.188 g range: 0.67–1.48 g	0.3720 g range: 0.31–0.46 g	0.71 g range: 0.55–0.78 g	0.6160 g range: 0.11–1.11 g	1.2420 g range: 0.55–1.99 g	0.2480 g range: 0.15–0.37 g	0.56 g range: 0.45–0.66 g
	Average TAMWR	0.5519 range: 0.357142857–0.729729729		0.5316 range: 0.397435897–0.63		0.4534 range: 0.2–0.6094674556		0.4458 range: 0.2878787878–0.6086956521	

**Table 3:** Histological evaluation of regenerated nerves.

	16 Weeks Control Group	21 Weeks Control Group	16 Weeks Study Group	21 Weeks Study Group
Average number of regenerated nervous fibers	59 ± 5	65 ± 3.9	61 ± 3	67 ± 2.6
Fiber area	4410 ± 150 μm^2^	4854 ± 150 μm^2^	4586 ± 148 μm^2^	5044 ± 130 μm^2^
Fiber density	0.0109 ± 0.0004 fibers/μm^2^	0.012 ± 0.0004 fibers/μm^2^	0.0112 ± 0.0005 fibers/μm^2^	0.0123 ± 0.0005 fibers/μm^2^

## Data Availability

All data generated or analyzed during this study are included in this published article.
